# Impact of Spacer Nature and Counter Ions on Rheological Behavior of Novel Polymer-Cationic Gemini Surfactant Systems at High Temperature

**DOI:** 10.3390/polym12051027

**Published:** 2020-05-01

**Authors:** Shams Kalam, Muhammad Shahzad Kamal, Shirish Patil, Syed Muhammad Shakil Hussain

**Affiliations:** 1 Department of Petroleum Engineering, King Fahd University of Petroleum & Minerals, Dhahran 31261, Saudi Arabia; g201306530@kfupm.edu.sa (S.K.); patil@kfupm.edu.sa (S.P.); 2 Center for Integrative Petroleum Research, King Fahd University of Petroleum & Minerals, Dhahran 31261, Saudi Arabia; smshakil@kfupm.edu.sa

**Keywords:** rheological properties, enhanced oil recovery, gemini surfactants, polymers, surfactant-polymer system

## Abstract

Compatible surfactant-polymer (SP) hybrid systems at high temperature are in great demand due to the necessity of chemical flooding in high-temperature oil reservoirs. The rheological properties of novel SP systems were studied. The SP system used in this study consists of a commercial polymer and four in-house synthesized polyoxyethylene cationic gemini surfactants with various spacers (mono phenyl and biphenyl ring) and different counterions (bromide and chloride). The impact of surfactant concentration, spacer nature, counterions, and temperature on the rheological features of SP solutions was examined using oscillation and shear measurements. The results were compared with a pure commercial polymer. All surfactants exhibited good thermal stability in seawater with no precipitation. Shear viscosity and storage modulus were measured as a function of shear rate and angular frequency, respectively. The experimental results revealed that the novel SP solution with a mono phenyl and chloride counterions produces a better performance in comparison with the SP solution, which contains mono phenyl and bromide counterions. Moreover, the effect is enhanced when the mono phenyl ring is replaced with a biphenyl ring. Shear viscosity and storage modulus decrease by increasing surfactant concentration at the same temperature, due to the charge screening effect. Storage modulus and complex viscosity reduce by increasing the temperature at a constant angular frequency of 10 rad/s. Among all studied SP systems, a surfactant containing a biphenyl ring in the spacer with chloride as a counterion has the least effect on the shear viscosity of the polymer. This study improves the understanding of tuning the surfactant composition in making SP solutions with better rheological properties.

## 1. Introduction

Primary, secondary, and tertiary recovery methods are the three broad categories of oil recovery mechanisms. Waterflooding is a widely applied secondary oil recovery technique that recovers approximately one-third (1/3) of the original oil in place [1]. Globally, approximately 2 trillion barrels of conventional oil and 5 trillion barrels of unconventional heavy oil is left inside the reservoirs after employing traditional methods to recover oil [2,3]. Enhanced oil recovery (EOR) techniques lie in the tertiary domain, used to unlock the remaining significant amount of oil. Numerous EOR techniques have been developed to recover more oil, such as gas injection, chemical methods, and thermal methods [4,5,6,7,8,9,10]. Miscible gas injection is an effective method for high-pressure reservoirs such as CO_2_ flooding [11]. Low-salinity waterflooding/smart waterflooding has shown improvement in the oil recovery from carbonate rocks at high temperatures because of wettability alteration [12]. Chemical Enhanced Oil Recovery (cEOR) methods such as a surfactant, polymer, alkali flooding, and a blend of these chemicals are found to be successful techniques to unlock more oil from carbonate reservoirs [13]. Surfactants decrease the interfacial tension (IFT) between oil and water phases, while polymers enhance the displacement efficiency by increasing the viscosity of the injected fluid, hence improving the mobility [13,14,15,16,17,18]. Recently, it has been proved that polymers having higher elasticity (storage modulus) improves the sweep efficiency. Thus, polymers also enhance microscopic sweep efficiency because of elasticity, in addition to macroscopic displacement efficiency [19,20,21,22,23,24,25]. The use of alkali increases the pH, which produces surfactants by reacting with the organic acid available in the crude oil and helps in minimizing the adsorption of surfactant [4,26]. On the other hand, there are some complications with the use of alkali, such as scale formation. Surfactant polymer (SP) flooding without the use of alkali can prevent complications associated with alkali usage and thus lowers the operating expenses for EOR projects [26]. The exploitation of nanotechnology in improving the efficiency of EOR techniques has been studied in recent years, consisting of numerous theoretical and experimental developments [27,28,29]. Several chemical EOR projects have been implemented across the globe, such as in China, the USA, and Canada. The largest field application of chemical EOR is found in China with a 300,000 bbl/day of oil increment [30]. 

Understanding the structure and nature of the SP system is very important when designing a successful cEOR formulation. Mainly, three kinds of interactions exist between surfactant and polymer: (1) attractive forces among molecules of surfactant are very low in comparison with repulsive forces between surfactant and polymer, (2) attractive forces between surfactant and polymer are lower than attractive forces among molecules of surfactant, (3) attractive forces between surfactant and polymer are greater than forces among surfactant molecules [31]. Gemini surfactants are a unique class of surfactants with more water-soluble hydrophilic head and oil-soluble hydrophobic tail units [32,33,34,35]. Both groups are connected by a spacer at or near to the head groups. Polymers are added into the surfactants to increase the sweep efficiency of the SP flooding process, as discussed earlier. Several studies were conducted to understand the behavior of the SP system, such as IFT, contact angle, adsorption, phase behavior, rheology, foaming, zeta-potential, and core flooding experiments [4,13,36,37,38,39,40,41,42,43]. 

The study of rheological parameters plays a significant role in screening and understanding various SP hybrid systems for cEOR operation. Several investigations have been carried out on the rheological behavior of polymers, surfactants, and the synergy of both. For instance; the study of rheological properties of scleroglucan and N-vinylpyrrolidone polymers [44], sodium surfactin [45], SP systems comprised of a copolymer of acrylamide and acrylamido tertiary butyl sulfonate and sodium dodecyl sulfate surfactant [46], and erucyl dimethyl amidobetaine viscoelastic surfactant and its hybridization with partially hydrolyzed polyacrylamide polymer [47]. 

**Figure 1:**
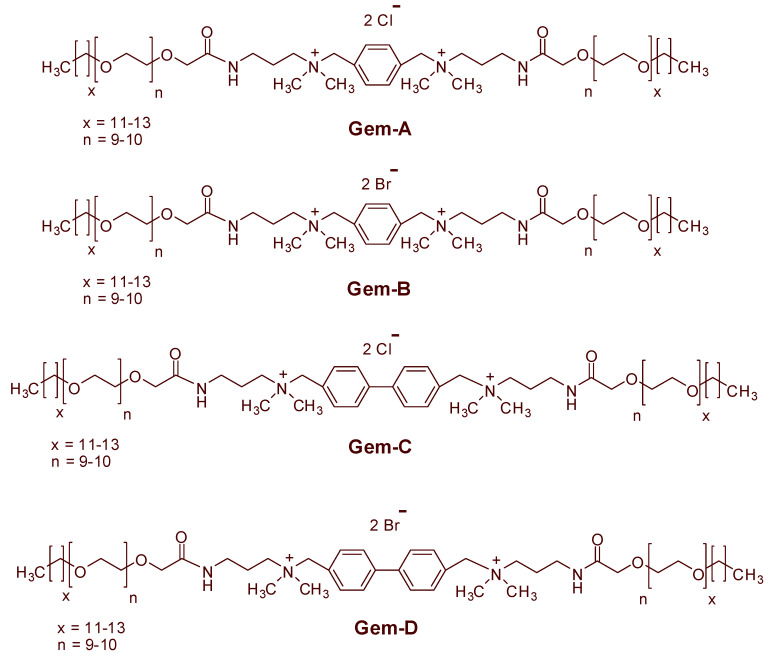
Structures of cationic gemini surfactants (Gem-A to Gem-D) used in this study.

This paper presents an extensive study of rheological properties of novel SP solutions considering the effect of surfactant concentration, temperature, spacer nature, and counterions. Four cationic gemini surfactants (Gem-A to Gem-D) were synthesized in the laboratory, each with similar head and tail groups, differing by the spacer group and counterions (Figure 1). For example, Gem-A contains a mono phenyl ring in the spacer with chloride counterions, and Gem-B possesses a mono phenyl ring in the spacer with bromide counterions. Similarly, Gem-C comprises of a biphenyl ring in the spacer with chloride counterions, and Gem-D contains a biphenyl ring in the spacer with bromide counterions. Likewise, four polyacrylamide based cationic polymers with different molecular weights were utilized (Table 1). Firstly, the rheological properties of four cationic polyacrylamides were presented. Secondly, the rheological properties of the proposed SP systems were discussed. This includes the effect of surfactant concentration, spacer nature, counterions, and temperature on rheological properties in detail.

## 2. Materials and Methods

### 2.1. Materials

Four different polymers and surfactants were used in this work. All polymers were supplied by SNF Floerger (Andrézieux, France). The concentration of the polymers was fixed at 2500 ppm. The cationic polyacrylamide (FO 4290 SSH) was used with different concentrations of newly synthesized cationic gemini surfactants for rheological measurements. The seawater used in this study was synthesized in the laboratory using sodium, calcium, magnesium, sulfate, chloride, and bicarbonate ions with total dissolved solids (TDS) of 57,670 ppm (mg/L). The composition of the laboratory-made seawater is shown in Table 2.

### 2.2. Preparation of Polymer Solutions

A 0.25 weight% polymer was added into the beaker containing 100 mL deionized water. Two hours of magnetic stirring and twenty hours of retention time at room temperature were deemed sufficient to make uniform polymer solutions and ensure complete hydration. During magnetic stirring, a polymer was gradually added into the beaker containing deionized water, on the shoulder of its vortex to prevent lumping. The concentration of polymer was kept constant for all solutions in this study.

### 2.3. Rheological Measurements

The rheological properties of novel SP solutions were determined using a Discovery Hybrid Rheometer (DHR-3) from TA Instruments (New Castle, DE, USA). The geometric configuration set for this work was a concentric cylinder. Rheological experiments were conducted in a temperature range from 20 °C to 80 °C, whereas, the shear rate for steady shear viscosity measurements ranged from 0.001 to 1000 s^−1^. Frequency sweep experiments were performed in the region of linear viscoelasticity. Shear viscosity (η) depicts the measure of resistance to flow, while storage modulus (G’) shows the amount of energy stored in the elastic material (i.e., material elasticity). Viscosity and storage modulus were measured as a function of shear rate (ϒ) and angular frequency (ω) respectively, at different surfactant concentrations, ranging from 0 to 0.5 mM, where 0 mM shows pure polymer. The effect of temperature was investigated at an angular frequency of 10 rad/s.

## 3. Results and Discussion

The discussion is split into two major parts. Rheological properties of cationic polyacrylamide are discussed in the first part. The second part discussed the rheological properties of the novel surfactant polymer (SP) systems.

### 3.1. Rheological Properties of Cationic Polyacrylamide

**Figure 2:**
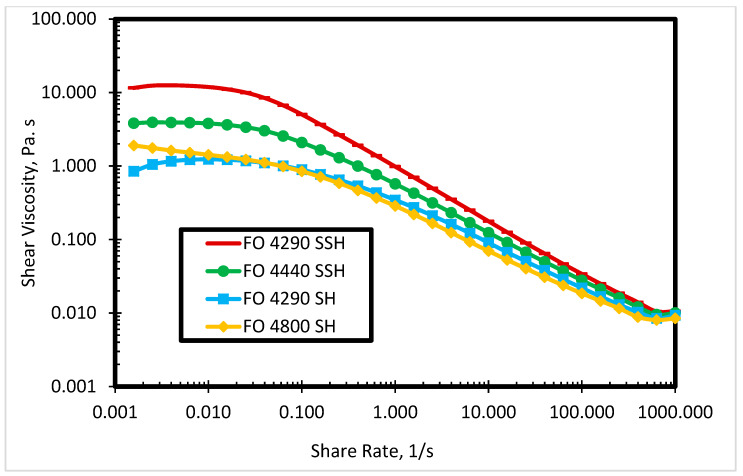
Shear viscosity of commercial polymers in deionized water.

**Figure 3:**
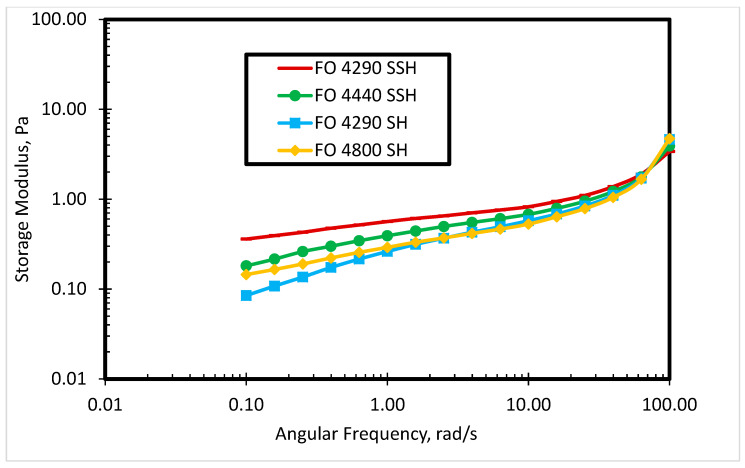
The storage modulus of commercial polymers in deionized water.

A rheological study of four commercial cationic polyacrylamides was carried out. The molecular weight of each polymer is shown in Table 1. Figure 2 depicts the steady shear viscosity of the polymer solutions in deionized water at 80 °C. The behavior in Figure 2 shows that shear viscosity is dependent on the molecular weight of the polymer. At a lower shear rate (<10 s^−1^), the high molecular weight polymer has a higher shear viscosity. However, at a higher shear rate, the difference in shear viscosity of the polymer solutions is reduced due to shear dependent viscosities of the polymers. From 0.001 to 0.1 s^−1^ shear rate, the shear viscosity is constant for each polymer solution, while the shear viscosity is linearly dependent on the shear rate after 0.1 s^−1^. Figure 3 shows the effect of storage modulus for different polymers at different angular frequencies. At lower angular frequency (<10 rad/s), the storage modulus is higher for the high molecular weight polymer solutions, as presented in Figure 3. Nevertheless, the difference in the storage modulus of polymer solutions is not significant at higher angular frequencies. FO 4290 SSH polymer was selected based on rheological study (highest shear viscosity and storage modulus among four polymer solutions) to combine with several newly synthesized cationic gemini surfactants to produce and analyze novel SP systems for the cEOR processes.

### 3.2. Rheological Properties of the SP System

The interaction of surfactants with polymers has a huge impact on the rheological behavior of SP systems, which is discussed in the following section.

#### 3.2.1. Effect of Surfactant Concentration

**Figure 4:**
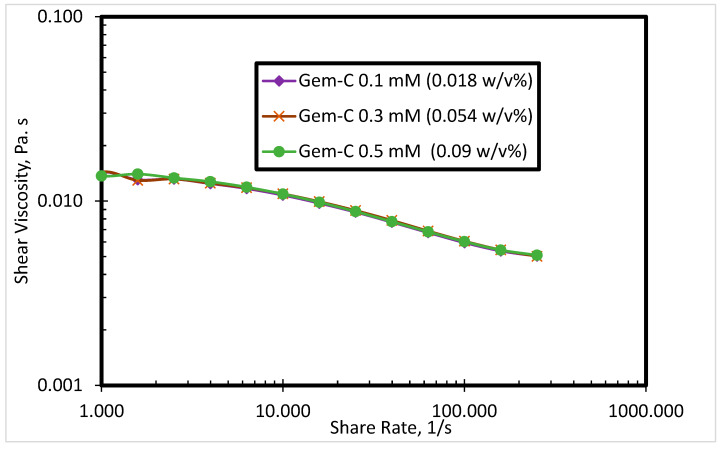
Shear viscosity of Gem-C at different concentrations in seawater.

The rheological behavior of the SP solutions having cationic gemini surfactants was evaluated at three different surfactant concentrations (0.1 mM, 0.3 mM, and 0.5 mM) at 80 °C. Four synthesized cationic gemini surfactants with different spacer nature and counterions are used. All surfactants exhibited good thermal stability in seawater with no precipitation. Figure 4 shows the shear viscosity of Gem-C at different concentrations in seawater. The results in Figure 4 show that the shear viscosity of Gem-C did not significantly change by increasing the surfactant concentrations in seawater. The precipitation of surfactants in the seawater was not seen.

**Figure 5:**
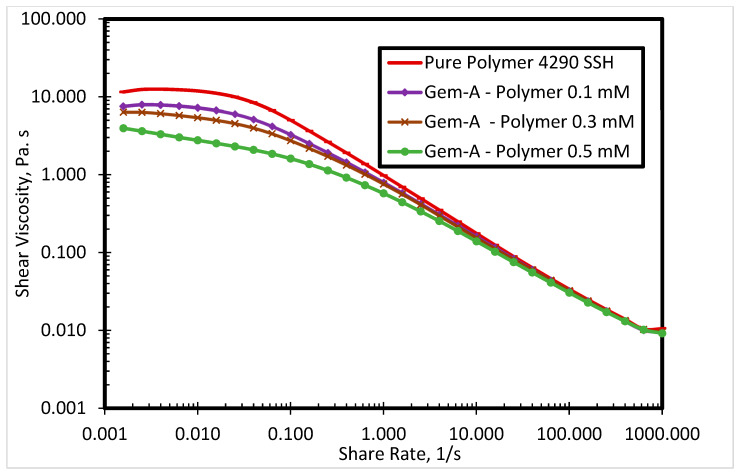
Shear viscosity of 4290 SSH polymer with Gem-A surfactant at different concentrations.

**Figure 6:**
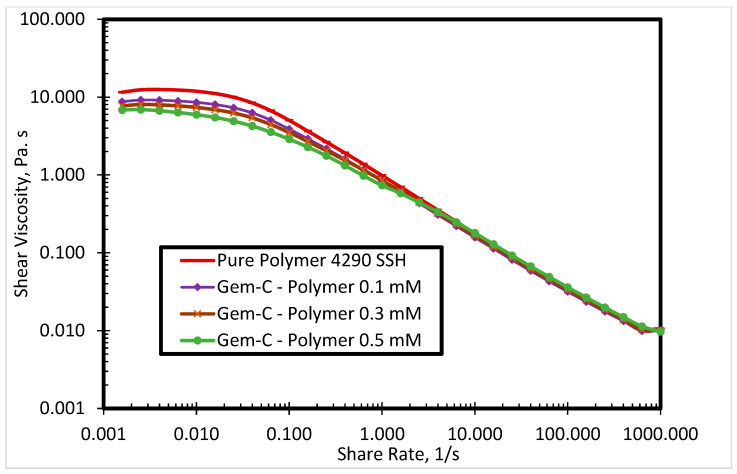
Shear viscosity of 4290 SSH polymer with Gem-C surfactant at different concentrations.

**Figure 7:**
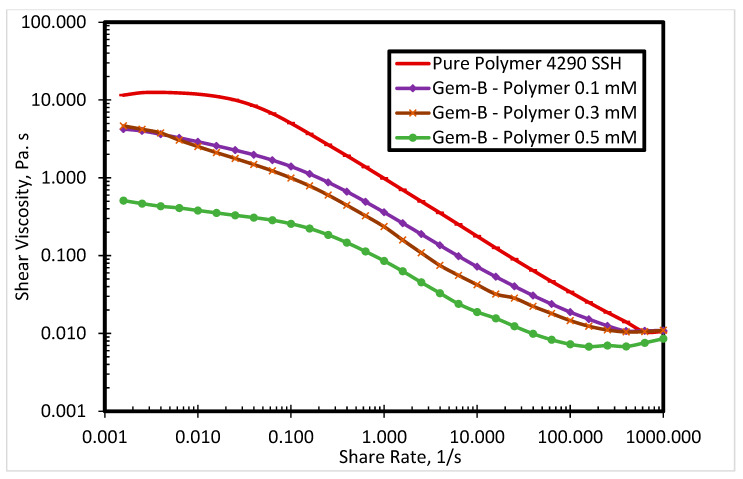
Shear viscosity of 4290 SSH polymer with Gem-B surfactant at different concentrations.

**Figure 8:**
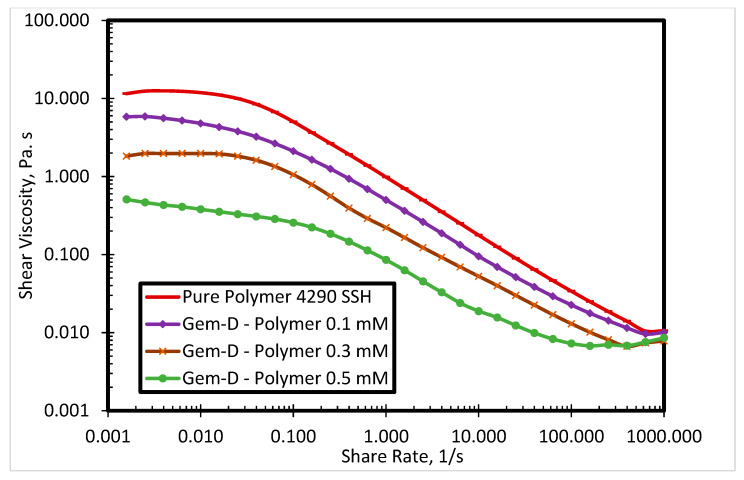
Shear viscosity of 4290 SSH polymer with Gem-D surfactant at different concentrations.

The commercial polymer FO 4290 SSH showed the highest viscosity among all evaluated SP systems. Thus, it was selected to investigate the surfactant-polymer interactions with newly synthesized surfactants. Figure 5 presents the impact of Gem-A surfactant concentration (0.1, 0.3, and 0.5 mM) on the shear viscosity of the SP system at 80 °C. By increasing the surfactant concentration, the polymer viscosity decreases. Both polymers and surfactants used in this research were cationic. The red curve in Figure 5 shows the polymer shear viscosity profile, which is highest among all curves. The higher shear viscosity of the pure polymer is due to the fact that it has no interaction with the surfactant. However, the addition of the surfactant decreases the viscosity of the polymer. The higher the concentration of the surfactant, the greater the reduction in polymer viscosity. A possible explanation for this behavior is that the interactions between anionic counterions of cationic surfactants and cationic polymers result in charge screening, which leads to lower polymer viscosity. Figure 5 shows the behavior of Gem-A, in which chloride is used as an anionic counterion. This reduction in viscosity is only significant at lower shear rates, while all solutions exhibit the same trend at higher shear rates. This is because of the impact of the high shear rate surpassing the charge screening effect. Similar behavior is observed for Gem-C at 0.1, 0.3, and 0.5 mM concentrations, as shown in Figure 6. Gem-B and Gem-D show different behaviors in the higher shear rate region at 0.1, 0.3, and 0.5 mM concentrations, as shown in Figure 7 and Figure 8, respectively. In the higher shear rate region, the SP system viscosity for Gem-B and Gem-D also decreases with increasing surfactant concentrations, unlike Gem-A and Gem-C. The percentage decrease of SP system viscosity is higher for Gem-B and Gem-D compared to Gem-A and Gem-C, as shown in Figure 5, Figure 6, Figure 7 and Figure 8. This means that the charge screening effect is higher in Gem-B and Gem-D.

**Figure 9:**
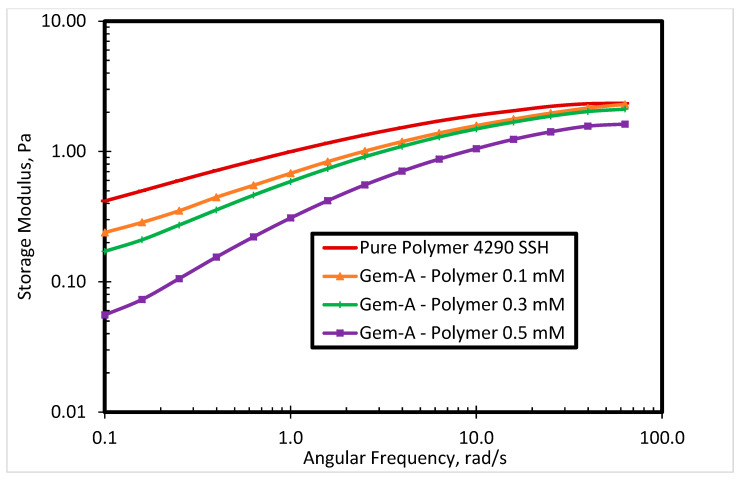
The storage modulus of 4290 SSH polymer with Gem-A surfactant at different concentrations.

Storage modulus shows material elasticity, which is an important property in the flooding processes. The impact of Gem-A concentration in SP solution on storage modulus is shown in Figure 9. The storage modulus of the polymer is decreased by adding the surfactant. The higher the surfactant concentration in the SP system, the lower the storage modulus. A possible explanation for this behavior is charge screening between cationic surfactant-containing anionic counterions and cationic polymers. This effect is significant at lower angular frequencies, while higher angular frequency dominates over the charge screening effect.

#### 3.2.2. Effect of Spacer Nature and Counterions

**Figure 10:**
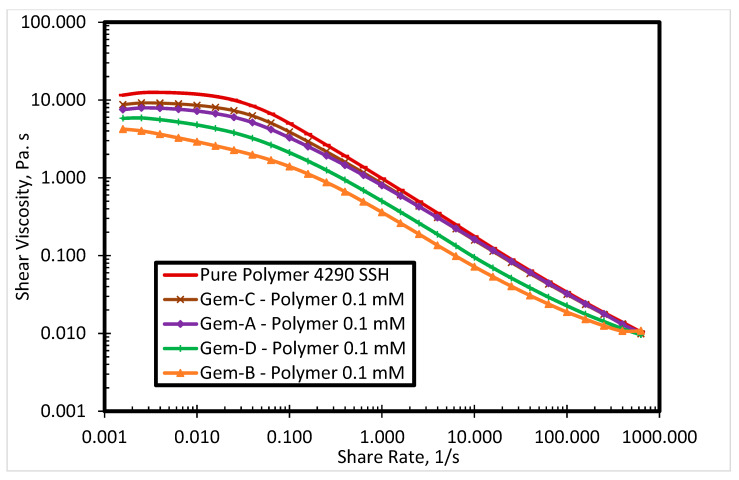
Shear viscosity of 4290 SSH polymer with all surfactants at 0.1 mM concentration.

**Figure 11:**
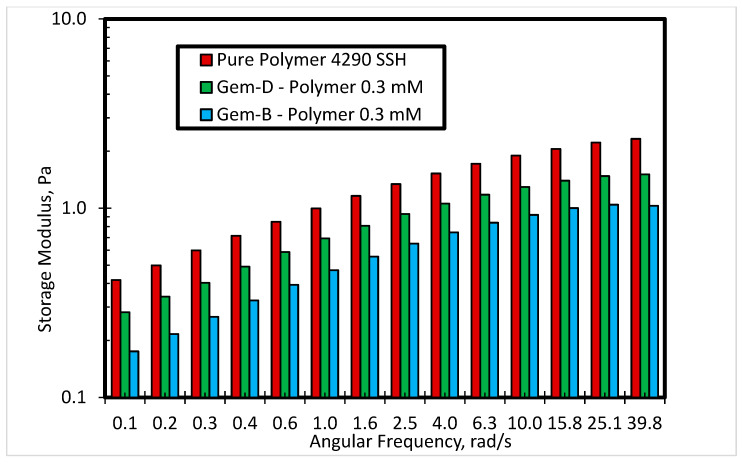
Storage modulus of 4290 SSH polymer with Gem-D and Gem-B at 0.3 mM concentration.

**Figure 12:**
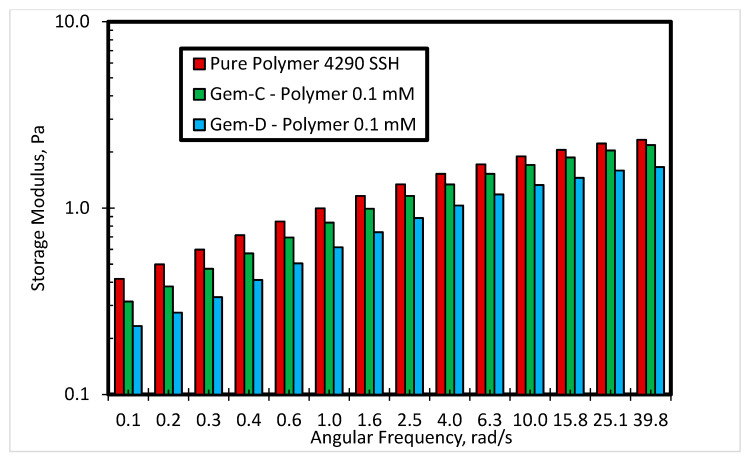
The storage modulus of 4290 SSH polymer with Gem-C and Gem-D at 0.1 mM concentration.

Figure 10 presents the effect of spacer nature and counterions on shear viscosity at constant surfactants’ concentration of 0.1 mM, while Figure 11 and Figure 12 show the impact of spacer nature and counterions on storage modulus at 0.3 and 0.1 mM surfactants, respectively. The intensity of viscosity reduction is higher for surfactants with bromide counterions as compared to the surfactant with chloride counterions (Figure 10). Gem-B and Gem-D contain similar counterions (bromide), but shear viscosity profile for Gem-D is above that of Gem-B, which reveals that viscosity reduction decreases by the addition of another phenyl ring in the spacer. A similar trend was also observed between Gem-A and Gem-C, but the difference was insignificant. Gem-B gives the lowest viscosity of the SP solution at 0.1 mM and 80 °C.

Figure 11 shows the effect of the phenyl ring in the spacer of the cationic gemini surfactant with bromide counterions on storage modulus at 0.3 mM concentration and 80 °C. The results show that the inclusion of another phenyl ring in the spacer can help in improving the elasticity of the material. Gem-D shows a higher storage modulus as compared to Gem-B at all angular frequencies. Figure 12 depicts the impact of counterions on storage modulus for cationic gemini surfactants containing biphenyl rings in the spacer at 0.1 mM concentration. Gem-C with chloride counterions shows a higher storage modulus as compared to Gem-D with bromide counterions at all angular frequencies, which means that the use of chloride counterions can help in enhancing the elasticity of the SP system.

#### 3.2.3. Effect of Temperature

**Figure 13:**
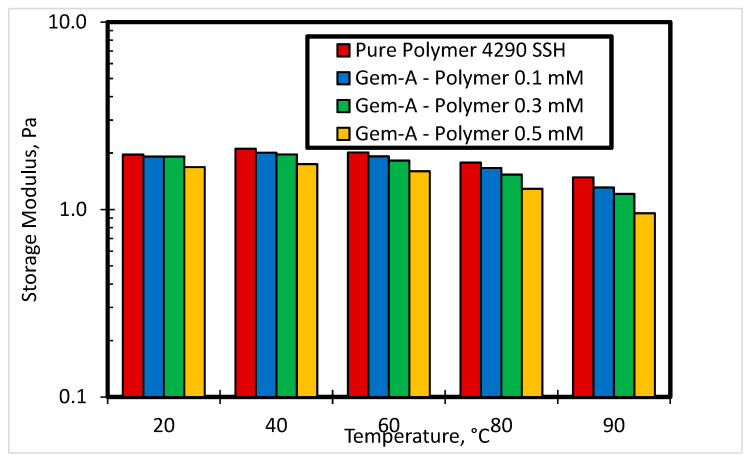
Effect of temperature on storage modulus at different concentrations of Gem-A in SP system at angular frequency 10 rad/s.

**Figure 14:**
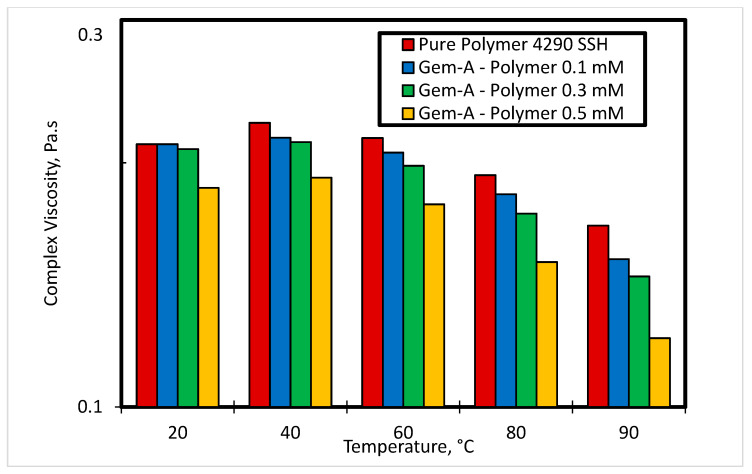
Effect of temperature on complex viscosity at different concentrations of Gem-A in the SP system at angular frequency 10 rad/s.

Figure 13 and Figure 14 show the effect of temperature at various concentrations of Gem-A in the SP system on storage modulus and complex viscosity, respectively. The angular frequency for these experiments was kept constant at 10 rad/s, and the temperature was varied from 20 °C to 90 °C. Increasing temperature results in a reduction in both storage modulus and complex viscosity, as shown in Figure 13 and Figure 14, respectively. Increasing temperature reduces the intermolecular forces of the material, and hence reduces the elasticity and complex viscosity. This effect enhances at higher surfactant concentrations.

## 4. Summary and Conclusions

An extensive rheological study was conducted on newly synthesized cationic gemini surfactants along with the cationic polymer. The study includes the effect of surfactant concentration, spacer nature, counterions, and temperature on shear viscosity and storage modulus using oscillation and shear measurements. This work further improves the understanding of tuning the surfactant structure in making SP solutions of required rheological properties. The following conclusions can be drawn from this study.

- An increase in surfactant concentrations decreases the viscosity and elasticity of the SP system because of the charge screening effect.
- The inclusion of the phenyl ring in the spacer can help in improving the viscosity and elasticity of the SP system.
- The use of chloride counterions can give better rheological behavior as compared to bromide counterions.
- Finally, it was observed that counterions also influence the rheological properties significantly. The results reveal that the novel SP solution with a phenyl ring with chloride counterions performs better in comparison to a phenyl ring with bromide counterions. Moreover, the performance of the SP solution system can be further enhanced by the addition of another phenyl ring in the spacer.

## Figures and Tables

**Table 1:** Molecular weights of cationic polyacrylamide.

Polymer Name	Molecular Weight (million g/mol)
**FO 4290 SSH**	8.3–10.8
**FO 4440 SSH**	6.8–9.1
**FO 4290 SH**	5.7–8.3
**FO 4800 SH**	4.7–6.7

**Table 2:** Chemical composition of laboratory-made seawater.

Ions	Seawater (ppm)
**Sodium**	18,300
**Calcium**	650
**Magnesium**	2110
**Chloride**	32,200
**Bicarbonate**	120
**Sulfate**	4290
**TDS**	57,560
